# Multiperiodic orbits from interacting soft spots in cyclically sheared amorphous solids

**DOI:** 10.1126/sciadv.abg7685

**Published:** 2021-08-11

**Authors:** Nathan C. Keim, Joseph D. Paulsen

**Affiliations:** 1 Department of Physics, Pennsylvania State University, University Park, PA 16802, USA.; 2 Department of Physics, California Polytechnic State University, San Luis Obispo, CA 93407, USA.; 3 Department of Physics, Syracuse University, Syracuse, NY 13244, USA.; 4 BioInspired Syracuse: Institute for Material and Living Systems, Syracuse University, Syracuse, NY 13244, USA.

## Abstract

When an amorphous solid is deformed cyclically, it may reach a steady state in which the paths of constituent particles trace out closed loops that repeat in each driving cycle. A remarkable variant has been noticed in simulations where the period of particle motions is a multiple of the period of driving, but the reasons for this behavior have remained unclear. Motivated by mesoscopic features of displacement fields in experiments on jammed solids, we propose and analyze a simple model of interacting soft spots—locations where particles rearrange under stress and that resemble two-level systems with hysteresis. We show that multiperiodic behavior can arise among just three or more soft spots that interact with each other, but in all cases it requires frustrated interactions, illuminating this otherwise elusive type of interaction. We suggest directions for seeking this signature of frustration in experiments and for achieving it in designed systems.

## Teaser

Small groups of hysteretic elements can explain how a system returns to a previous state only after multiple driving cycles.

## INTRODUCTION

**Fig. 1:**
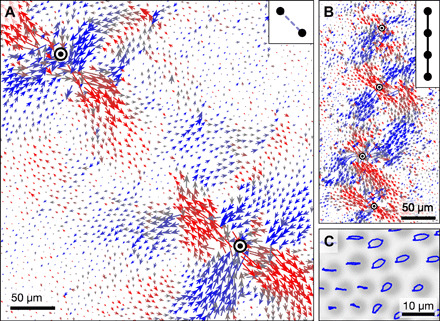
Interacting soft spots and periodic orbits in experiments on a cyclically sheared 2D jammed solid. (**A**) Particle displacements around two rearranging soft spots (approximate centers marked with ⊙) undergoing horizontal shear. Colors denote displacements along the two principal axes of shear. The displacements oppose each other at the center of the panel, suggesting a frustrated interaction. Inset: Schematic of the frustrated interaction (dashed lines). (**B**) Displacements around a group of several soft spots, suggesting cooperative interactions. Inset: Schematic of cooperative interactions (solid lines). (**C**) Steady-state particle paths, which are closed with the same period as the driving. Multiperiodic paths would have a longer period. Background: Experimental micrograph.

A solid with perfectly elastic behavior deforms reversibly, in the sense that all material points return to their initial positions when a load is removed. Some amorphous solids may be prepared in a reversible plastic state, wherein loading the material in one direction changes its structure through many microscopic events, but loading it in the reverse direction precisely undoes these changes (*1*–*5*). Each microscopic event is localized to a soft spot (*6*) or shear-transformation zone (Fig. 1A) (*7*), which resembles a two-level system that switches under forward and reverse shear (*4*, *7*–*9*).

Recent simulations using athermal quasi-static shear have revealed an even more remarkable behavior in which the period of particle motions is a multiple of the period of driving (*1*, *10*), reminiscent of the familiar action of a retractable pen. Such “multiperiodic” behavior may sound quite tenuous, given the daunting number of mechanically stable configurations and transitions in a packing of even a modest size. Nevertheless, multiperiodicity has been observed in molecular dynamics simulations of amorphous solids in two and three dimensions for several kinds of particle interactions (*1*, *10*–*15*). However, the mechanism for this behavior has remained unclear, even as it seems to be associated with an unjamming transition as the confining pressure is decreased (*13*).

**Fig. 2:**
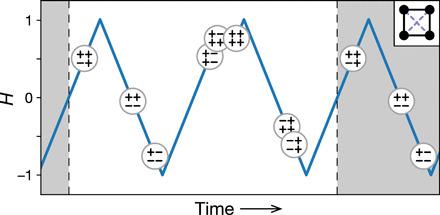
Example of a *T=*2 orbit in our model of interacting hysterons. Inset: Arrangement of the four hysterons with a mixture of cooperative and frustrated interactions (solid and dashed lines, respectively). Main: Each large circle represents a state of the system and is placed at the value of external field *H* at which the system reaches that state. The close pair of states near *H* = 1 constitutes an avalanche. The time axis indicates only the sequence of events, since the simulation is quasi-static.

Here, we show how multiperiodicity can arise in a simplified coarse-grained model of interacting soft spots (Fig. 2). We identify how the prevalence of multiperiodicity depends on the spatial arrangement of the soft spots, and we show how to design the behavior on demand. In all cases, the multiperiodic orbits are made possible by frustrated interactions in our model. Our results show that frustrated interactions between soft spots must be considered as an important counterpart to the cooperative interactions that are used to explain avalanches near the yielding transition (*5*, *16*, *17*).

## RESULTS

While experiments have not yet observed multiperiodic behavior, they exhibit the microscopic phenomenology we wish to distill into our model. Figure 1A shows a displacement field from an experiment with two nearby soft spots (see Materials and Methods for details). Each has the characteristics of an Eshelby inclusion—a small region of plastic deformation that is coupled to a quadrupolar elastic deformation of the surrounding material. This extended deformation induces or inhibits the rearrangement of other nearby soft spots, depending on their relative placement (*8*, *16*, *18*). For example, Fig. 1A is suggestive of a frustrated interaction, whereas the arrangement in Fig. 1B suggests cooperative interactions.

Our jumping-off point is to consider the possible behaviors of compact collections of *N* soft spots by modeling them as interacting hysteretic elements or “hysterons” (*8*, *9*). A hysteron has two possible states, *s_i_* = ±1; it transitions to the “+” state when the local field—equal to the instantaneous global strain field *H* plus neighbor interactions—reaches a fixed threshold $H_{i}^{+}$. Likewise, it transitions to the “−” state at a fixed threshold $H_{i}^{-}<H_{i}^{+}$. To model the disorder of such packings, these thresholds are set as $H_{i}^{+}=h_{i}+u_{i}$ and $H_{i}^{-}=h_{i}-u_{i}$, where *h_i_* is chosen with uniform probability from the interval [ −1,1], and *u_i_* is chosen from [0, 2], for each hysteron independently. Hysteron *j* imposes a local field on hysteron *i* equal to *J_ij_s_j_*, where the coupling strength *J_ij_* is taken to be symmetric (*J_ij_* = *J_ji_*) except where stated otherwise. The magnitude of each *J_ij_* (with *i* ≠ *j*) is selected with uniform probability so that ∣*J_ij_* ∣ ≤ 1.

To capture the effect of the characteristic quadrupolar elastic deformations of rearranging soft spots, the signs of the *J_ij_* are dictated by the spatial configuration of the hysterons. Pairs that are 45° off the shear direction have a frustrated coupling (antiferromagnetic, *J_ij_*, *J_ji_* < 0), whereas pairs along 0° or 90° have a cooperative coupling (ferromagnetic, *J_ij_*, *J_ji_* > 0). This rule assumes that all soft spots’ displacement fields have approximately the same orientation and polarity relative to the direction of shear, which appears to be true broadly in experiments (*2*, *4*, *9*, *18*).

Our simulations, available as an open-source Python package (*19*), probe the system evolution under athermal, quasi-static, oscillatory driving between −*H*_0_ and +*H*_0_. We initialize the system with *H* ≪ −1 and all hysterons negative (*s_i_* = −1), and we evolve forward using an event-based method. Since flipping one hysteron may prompt a neighbor to flip, we wait for avalanches at fixed field until a stable state is reached; the hysteron farthest past its threshold is flipped first and all the local fields are updated between flips. In extremely rare cases where no stable state can be found or two flips are degenerate, the system is discarded. We continue driving until an absorbing state is reached where the dynamics repeat under further driving.

To search for multiperiodic behavior efficiently given the couplings *J_ij_* and thresholds $H_{i}^{\pm }$, we note that increasing the driving amplitude *H*_0_ will not change the dynamics until it is large enough to cause an additional hysteron to flip. Therefore, a finite set of *H*_0_ will exhaust all possible dynamics under symmetric driving. To obtain this set, for each of the 2*^N^* possible states, we compute the two values of *H* that bound the interval of stability for the state. We then sort the list of absolute values of these *H* and take the midpoints between successive values as our set of *H*_0_. We perform a series of simulations starting with the smallest *H*_0_ and continuing until any multiperiodic orbit is found. Such an “amplitude sweep” is likewise an efficient method to search for unfamiliar behavior in experiments.

### Comparing arrangements of hysterons

Figure 2 shows an example of a multiperiodic orbit that is achieved for *N* = 4 hysterons arranged on a square. The system cycles through eight states over two driving periods, repeating this sequence indefinitely thereafter. This is just one possible *T* = 2 orbit for this spatial arrangement of *N* = 4 hysterons; it occurs with probability *P* = 8.37 × 10^−6^ (allowing permutation of hysterons and inversion of the $H_{i}^{\pm }$).

**Fig. 3:**
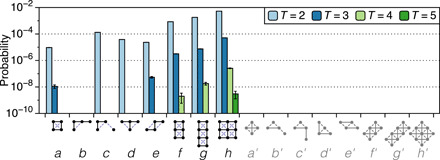
How the prevalence of multiperiodicity depends on the spatial arrangements of the hysterons. Probabilities of orbits with period *T* = 2,3,4,5, within the parameter space searched (*J_ij_* = *J_ji_*), for eight arrangements of hysterons *a* to *h*. Arrangements *f* to *h* exhibit a greater variety of periods and are consistent with an exponential decrease in probability as a function of the period (a straight line on these axes). In the diagrams along the *x* axis, solid (dashed) lines represent cooperative (frustrated) interactions with *J_ij_* > 0 (*J_ij_* < 0). For the complementary arrangements *a*′ to *h*′, no multiperiodic behavior was found. Error bars represent 68% confidence intervals; top′ bounds on zero probabilities (omitted from the plot for visual clarity) are 1.3 × 10^−9^ (*36*).

Figure 3 shows the prevalence of multiperiodicity in this and other compact arrangements of hysterons. The arrangements labeled *a* to *e* show all the unique configurations where *N* = 4 hysterons are placed within a 2 × 3 lattice that is oriented with the shear direction (up to reflections and rotations by 90°, which do not change the interactions). As before, interactions are between all nearest-neighbor pairs. Arrangement *c* has the highest probability of *T* = 2 among this set. These arrangements are some of the simplest ones eliciting multiperiodicity in our model.

Arrangements *f* to *h* in Fig. 3 show the increasing prevalence of multiperiodicity for *N* = 6, 8, and 9 hysterons on a square lattice. Arrangement *h* is multiperiodic with *P* = 5.3 × 10^−3^, so that if a macroscopic amorphous solid has 20 of these configurations, it will have a ∼10% chance of multiperiodicity. Notably, in contrast to the observed behavior of amorphous systems of many particles (*1*, *3*, *13*, *20*), small clusters of soft spots reach periodic orbits after very few cycles: For arrangement *h*, despite the space of 2^9^ states, the longest observed transient before a (multiperiodic) limit cycle was just 3 cycles, and it occurred in just 1 of 10^7^ systems.

When the lattice is rotated by 45° (exchanging cooperative and frustrated interactions, i.e., *J_ij_* → −*J_ij_*), no multiperiodic orbits are observed (arrangements *a*′ to *h*′). This curious observation leads us to note another special property of *a*′ to *h*′: If we assign a + or − state to any one hysteron, we can then work outward and assign states to all other hysterons, satisfying every interaction. This is because these arrangements are portions of an antiferromagnetic lattice, with ordered ground states. While it is unclear why this property might suppress multiperiodic behavior, it could be a starting point for a deeper understanding of multiperiodicity generally.

The above results demonstrate that multiperiodic orbits can arise in our simple model constructed from coupled hysterons. In the following sections, we identify which attributes of the model are necessary for producing multiperiodicity.

### Minimal number of hysterons

Empirically, we find that multiperiodic behavior is impossible for *N* < 3 hysterons. *N* = 3 hysterons with symmetric couplings also do not exhibit multiperiodic behavior. However, breaking the symmetry of at least one interaction pair (*J_ij_* ≠ *J_ji_*) is enough to allow a *T* = 3 orbit, if and only if all interactions are frustrated. Under these conditions, we observe *T* = 3 with *P* = 4.67 × 10^−3^, with a single unique sequence of states (see the Supplementary Materials). We observe *T* = 2 with *P* = 7.80 × 10^−3^, accounting for a variety of different sequences.

Asymmetric couplings in spin systems without external cyclic driving (*21*–*23*) have been studied before, but the physical meaning in a driven amorphous solid is unclear. One possible mechanism might be for soft spots to change states on different time scales, so that when the system is driven at finite frequency, a “slow” hysteron could fail to change in part of the cycle, even when in strict terms it is unstable.

### Role of frustration

**Fig. 4:**
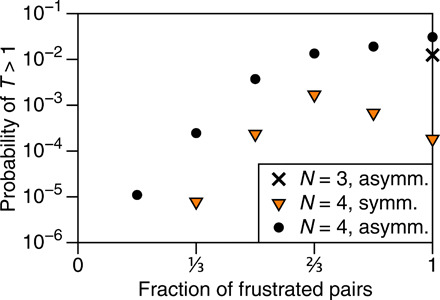
Occurrence of multiperiodic behavior for few hysterons. Probability is plotted as a function of the fraction of interaction pairs that are frustrated for symmetric (*J_ij_* = *J_ji_*) and asymmetric interactions. Error bars are smaller than the symbols. Multiperiodic behavior becomes exponentially less common as the fraction of frustrated pairs is reduced from ^2^/_3_ down to 0, but with no observed multiperiodicity in all 10^8^ systems when there are no frustrated pairs. (A straight line on these axes corresponds to an exponential trend.) For *N* = 3 with symmetric interactions, we observed no multiperiodic orbits at all.

The observation that all interactions must be frustrated to elicit multiperiodic behavior for *N* = 3 prompts us to further investigate the role of frustration. In Fig. 4, we vary the fraction of interaction pairs that are randomly chosen to be frustrated (*J_ij_*, *J_ji_* < 0), and we plot the prevalence of multiperiodicity under these conditions. There is a clear trend across all the data: Multiperiodic behavior becomes exponentially more scarce as the fraction of frustrated pairs is reduced from ^2^/_3_ down to 0. In all cases, the probability is identically zero in the absence of frustration, a result we have checked up to *N* = 7. Figure 4 also confirms that the topology of frustrated and cooperative interactions can be just as important as their number: Arrangement *a*′ in Fig. 3 has *N* = 4 and ^2^/_3_ of pairs frustrated, and yet we find no multiperiodic orbits for that specific topology for either symmetric or asymmetric interactions.

### Multiperiodicity from nonhysteretic elements

The above results show how coupled hysterons can produce multiperiodic orbits. We now show that hysteresis of the elements is not a necessary ingredient for multiperiodicity. In the absence of hysteresis and when −1 < *J_ij_* = *J_ji_* < 1, our model of an amorphous solid reduces to a spin glass where each soft spot corresponds to an Ising spin, governed by the Hamiltonian

$$H=-\frac{1}{2}\sum_{i\neq j}J_{\mathrm{ij}}s_{i}s_{j}-H\sum_{i}s_{i}$$ (1)

We verified this by writing separate code for such a spin glass and comparing the results with our coupled hysteron code with zero hysteresis. Deutsch and Narayan (*24*) reported multiperiodic orbits in such spin glasses with as few as five spins, although they focused on larger systems (*N* ≥ 64). We now elucidate the conditions for multiperiodicity with *N* = 5, under additional conditions that simplify the interactions even further: All the spin couplings are antiferromagnetic (*J_ij_* ≤ 0), and one or more of the couplings are randomly set to zero.

**Fig. 5:**
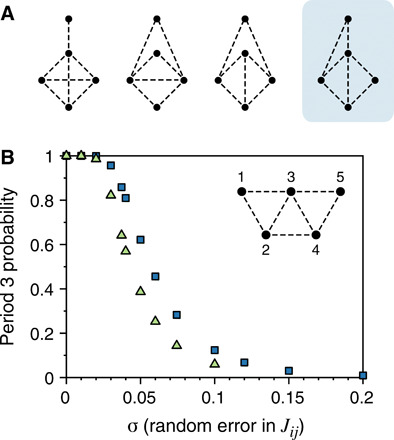
Multiperiodicity from frustrated nonhysteretic elements. (**A**) The four distinct graphs on *N* = 5 vertices with three missing edges. Multiperiodic orbits were found only in the rightmost graph, redrawn in (B) as a portion of a triangular lattice. (**B**) Probability of period 3 near the Chebyshev center of the period 3 polytope for the spin model in the inset, with *J*_12_ < *J*_45_ to lift a degeneracy. Squares: Probability of falling within the polytope for 10^6^ Gaussian-distributed points around the Chebyshev center while keeping *J*_14_ = *J*_25_ = *J*_15_ = 0. Triangles: All *J_ij_* are given random errors. In this case, the seven-dimensional description does not apply; one would need to characterize a distinct 10-dimensional polytope. Instead, 10^4^ simulations are run for each σ.

With four couplings set to zero, no multiperiodic orbits were observed in 10^6^ systems. With 3 couplings set to 0, of 10^7^ systems, we observe multiperiodicity in 1932—all with period *T* = 3 and a unique topology of interactions. This topology is shown in Fig. 5A and in the inset to Fig. 5B as a portion of a triangular lattice. Without loss of generality, we break the mirror symmetry by requiring ∣*J*_34_∣ < ∣*J*_01_∣ when spins are indexed left to right. This leads to an additional remarkable uniqueness: At the smallest *H*_0_ for multiperiodicity in each system, there is a unique and highly symmetric steady-state orbit (see the Supplementary Materials).

### Regions in parameter space

The evolution of this spin glass model is deterministic given the coupling strengths *J_ij_*, an initial condition, and a driving protocol. Working in the reverse direction, a sequence of states may be mapped back to a region of the (high-dimensional) space of *J_ij_* that can give this sequence; here, a subset of the unit hypercube [ −1,0]*^n^*, where *n* = 7 is the number of nonzero couplings. Proceeding in this manner, we find a set of 10 inequalities among the *J_ij_* that bound the region of parameter space corresponding to this *T* = 3 orbit, which we list in the Supplementary Materials. The volume of this high-dimensional polygon (i.e., polytope) is found to be 1.86 × 10^−4^.

To convert to a probability for multiperiodicity, we multiply this volume by 2 for the indexing degeneracy we lifted, and by ^1^/_2_ to account for the probability of obtaining the correct network topology. The latter factor may be found by noting that at least two of the three removed edges must share a vertex and enumerating the remaining cases. Thus, the above value is precisely the predicted probability of multiperiodic behavior for some *H*_0_. It agrees with how often we observe *T* = 3 in our simulations: *P* = (1.93 ± 0.04) × 10^−4^.

Such a detailed characterization of the high-dimensional phase space of the *J_ij_* is useful for designing systems with robust multiperiodic behavior. For instance, we can compute (*25*) a Chebyshev center for this polytope—a point that is farthest from its faces, which can thus withstand the largest possible errors in *J_ij_* while remaining multiperiodic. We find a Chebyshev center at

$$J=[\begin{array}{ccccc} 0 & -0.926 & -0.370 & 0 & 0\\ -0.926 & 0 & -0.519 & -0.278 & 0\\ -0.370 & -0.519 & 0 & -0.703 & -0.297\\ 0 & -0.278 & -0.703 & 0 & -0.703\\ 0 & 0 & -0.297 & -0.703 & 0 \end{array}]$$ (2)

which is the center of a hypersphere of radius 0.074 that lies entirely within the polytope. We report these coordinates to illustrate that our method can give precise quantitative information about finite regions of phase space that share a common orbit. At these coordinates, *T* = 3 is attained for any *H*_0_ in the range 1 < *H*_0_ < 1.685 (see the Supplementary Materials). For the general case of normally distributed errors in *J_ij_*, Fig. 5B shows that the probability of *T* = 3 remains high for an SD σ up to several hundredths. Thus, the low probability of multiperiodicity in this system stems from the enormity of the parameter space rather than a need for fine-tuning.

This same methodology—starting from an orbit and working backward to a region of parameter space—also applies to our model of interacting hysteretic soft spots. For example, setting *H*_0_ = 1, a Chebyshev center for the orbit in Fig. 2 is

$$J=[\begin{array}{cccc} 0 & -0.552 & 0.081 & 0.081\\ -0.552 & 0 & 0.670 & 0.280\\ 0.081 & 0.670 & 0 & -0.571\\ 0.081 & 0.280 & -0.571 & 0 \end{array}]$$ (3a)

$$H^{+}=[\begin{array}{cccc} 0 & -0.105 & 0.762 & 0.953 \end{array}]$$ (3b)

$$H^{-}=[\begin{array}{cccc} -0.809 & -0.220 & -0.856 & -1.047 \end{array}]$$ (3c)

which is a distance 0.081 from the nearest face. For the unique *T* = 3 orbit with *N* = 3 hysterons, a Chebyshev center is

$$J=[\begin{array}{ccc} 0 & -0.586 & -0.172\\ -0.172 & 0 & -0.586\\ -0.586 & -0.172 & 0 \end{array}]$$ (4a)

$$H^{+}=[\begin{array}{ccc} 0.828 & 0.828 & 0.828 \end{array}]$$ (4b)

$$H^{-}=[\begin{array}{ccc} -0.828 & -0.828 & -0.828 \end{array}]$$ (4c)

which is a distance 0.338 from the nearest face. Note the high degree of symmetry at this Chebyshev center: Each hysteron has identical *H*^+^ and *H*^−^, with identical asymmetric couplings that set up a clear chirality in the system. In the Supplementary Materials, we further characterize all of the above polytopes and list the inequalities that bound them.

## DISCUSSION

We have shown how multiperiodicity can arise from the interactions of a small number of localized soft spots with simple, physically motivated interactions. Previous studies of this behavior using molecular dynamics simulations did not consider localization to soft spots (*1*, *10*–*15*), while previous attempts to understand it using simplified models (*26*–*28*) did not pursue a microscopic picture of the system, e.g., of the sequence or spatial structure of rearrangements. In this work, by focusing on small systems, probing the effect of the spatial structure of the elements, and using an amplitude sweep for the driving field, we have provided a concrete and thorough foundation for addressing the origin of multiperiodicity in amorphous solids, where its robust appearance in simulations has not been well understood. While we have not specialized to particular distributions of parameter values that are a subject of current research (*17*, *29*), our general model is nevertheless able to capture a detail of the multiperiodicity found in molecular dynamics simulations: We observe an approximately exponential decay of probability with the period of the limit cycle, *T* (e.g., in arrangements *f* to *h* in Fig. 3), a trend that was reported by Lavrentovich *et al*. (*13*) in simulations on jammed solids. The findings of Lavrentovich *et al.* that multiperiodicity may be associated with an unjamming transition prompt the question of the role of soft spot interactions in this critical transition.

Our results show that frustrated interactions are always necessary for multiperiodic behavior. This comports with existing theory about the random-field Ising model (*30*), which showed that without frustration, it supports return-point memory—a behavior that precludes a multiperiodic response. These findings suggest that multiperiodicity should be taken as a conspicuous signature of frustration—a counterpart to the yielding and shear-banding behaviors that are often attributed to cooperative interactions (*5*, *16*, *17*).

Our results also offer guidance to experiments searching for multiperiodicity in amorphous solids. Because interactions among soft spots are crucial, strain amplitudes should be large enough to ensure a high density of switching soft spots but small enough to allow a periodic steady state—consistent with results of prior simulations that we can now rationalize with our model. Such experiments also promise to reveal the role of soft spot interactions near yielding (*8*, *17*) and to probe the limits of the return-point memory behavior that is incompatible with frustration (*8*, *9*, *30*–*32*). However, experiments must overcome measurement error and a high susceptibility to mechanical noise in this regime (*4*, *9*). We have shown that relatively few soft spots are sufficient for multiperiodic behavior, so that localized clusters of soft spots may be the dominant way that multiperiodicity emerges in large systems. Dividing observations of a large experimental system into regions of 𝒪(10) soft spots could thus enhance sensitivity to multiperiodic orbits while rejecting the effects of mechanical noise or initial conditions playing out elsewhere. Furthermore, it would test the hypothesis that multiperiodic behavior is highly localized rather than being a strictly emergent behavior spread out among many interacting particles. Combinations of small groups with incommensurate periods may be a way for longer-period orbits to arise.

We have also shown that specific multiperiodic behaviors among spins and hysterons correspond to convex regions in high-dimensional parameter space, bounded by systems of inequalities. This both serves as an additional check of our modeling and paves the way for the rational design of systems with these behaviors, for example, as the basis for a digital counter. Most promising are the *N* = 5 spin configuration (Fig. 5, inset) and the *N* = 3 and *N* = 4 hysteron configurations (Figs. 2 to 4), each of which is conducive to a real-space physical implementation, with network topology and bond strengths that might be realized in the lab.

## MATERIALS AND METHODS

### Details for Fig. 1

The experimental particle trajectories and micrograph used for Fig. 1 were obtained using methods described in (*9*), by cyclically shearing a monolayer of bidisperse polystyrene particles adsorbed at an oil-water interface. Because these particles exhibit long-range electrostatic repulsion (*33*), the material is a disordered, frictionless soft solid. We shear each sample between parallel boundaries that are 1.5 mm apart and 18 mm long; the material extends far beyond the open ends of this working sample. We image an approximately 1.4 × 1.9 mm region within the working sample. In fig. S1, we show micrographs corresponding to Fig. 1(A and B).

Each material is prepared by combining small and large sulfate latex microspheres (Invitrogen) in suspension, in roughly equal number, and dispersing them at an oil-water interface (*9*). The small and large particles in Fig. 1A have average diameters of 3.8 and 5.2 μm, and the particles in Fig. 1(B and C) have average diameters of 3.5 and 5.4 μm, although it is ultimately each particle’s electric dipole strength that determines its effective size in the packing (*33*). Although aggregates of several particles can form during the preparation process, they do not seem to be strongly correlated with the locations of soft spots.

We obtained the plotted displacements (Fig. 1, A and B) by comparing the position of each particle at two different times and subtracting the average motion of the region of surrounding material with radius 16.5*a*, where *a* is the mode of the interparticle distance, determined from the pair correlation function *g*(*r*) (*4*, *9*, *34*). We chose times when the shear strain γ = 0 (the midpoint of shearing), one full cycle apart. Figure 1A shows displacements in a portion of the system upon switching from strain amplitude 0.038 to 0.055. Figure 1B shows displacements in a different experiment upon switching from strain amplitude 0.045 to 0.050.

To obtain the plotted trajectory loops (Fig. 1C), we used positions over a full cycle of shearing at strain amplitude 0.055. Rather than subtracting the average motion within the region shown, we subtract the motion of a set of particles centered ∼35 μm below this region, so that the particles in the field of view appear to be displaced horizontally by the global shearing motion.

### Inequalities for regions of parameter space

For a system of spins, inequalities that bound regions of parameter space may contain only the parameters *H*_0_ and *J_ij_* as variables. To generate such inequalities from a sequence of states, we follow a method that parallels our simulation algorithm. Two examples illustrate our approach. We first consider a spin *i* that flips to the + state as *H* is increased. At this instant, the spin has become marginally unstable, so that

$$H+\sum_{j\neq i}J_{\mathrm{ij}}s_{j}=0$$ (5)

At this same instant, the other spins are stable, since otherwise they would have flipped before spin *i* did. For instance, if spin *k* ≠ *i* is in the − state,

$$H+\sum_{j\neq k}J_{\mathrm{kj}}s_{j}<0$$ (6)

where we use the previous states *s_j_* of the spins before spin *i* flipped. Substituting Eq. 5 into Inequality 6 yields an inequality that contains only the unknowns *J_ij_*, as desired. As a second example, we imagine that the flipping of spin *i* causes another spin *l* to flip immediately (an avalanche). This tells us not only that spin *l* is unstable at the same value of *H* given by Eq. 5 but also that, at that instant, it is farther past its threshold of stability than every other spin. The avalanche ends when all spins are stable; this observation leads to further inequalities by again combining Eq. 5 and Inequality 6 (where Inequality 6 is flipped for spins in the + state). A similar method applies to a system of hysterons, with $H_{i}^{+}$ and $H_{i}^{-}$ as additional unknowns on the right-hand side of Eq. 5 and Inequality 6 as needed. In general, additional inequalities are needed to denote that ∣*H* ∣ ≤ *H*_0_ at all times.

The resulting inequalities define a high-dimensional polygon (polytope). We use the Python package *pycddlib* (based on CddLib) to remove any redundant inequalities and the *pypoman* package (*25*) to compute Chebyshev centers. The full sets of inequalities for select orbits, further characterizations of the polytopes, and checks of the inequalities against our simulation results are given in the Supplementary Materials.

### Period 3 polytope volume in the spin model

To compute the volume of the period 3 polytope for *N* = 5 spins, we first convert from a set of inequalities to a set of vertices, using the Python package *pycddlib*. We then compute the volume of the convex hull of these points with the SciPy module *spatial.ConvexHull*. We find it to be 1.863 × 10^−4^. Measuring this volume using Monte Carlo integration with 10^8^ points gives consistent results: (1.857 ± 0.010) × 10^−4^. The full set of inequalities defining the polytope, and the 14 vertices they define, is given in the Supplementary Materials.

### Organizing the hysteron simulation orbits

Comparing orbits lets us meaningfully group and count systems with equivalent orbits. We represent each simulation’s output as a directed cyclic graph of states and manipulate it with the NetworkX package (*35*). We obtain the orbit by extracting the longest simple cycle in this graph. This removes trivial excursions: For instance, a system may transition from state + − + to + + + as *H* is increased and then return directly to + − + as *H* is decreased; we generally find many other randomly generated systems in which this excursion is missing. To compare these extracted orbits, we then account for all possible permutations of hysterons’ identities, reversal of the sequence, and inversion of the system (exchanging all the + and − states).

## SUPPLEMENTARY MATERIALS

Supplementary material for this article is available at http://advances.sciencemag.org/cgi/content/full/7/33/eabg7685/DC1
